# Potential miRNA biomarkers for the diagnosis and prognosis of esophageal cancer detected by a novel absolute quantitative RT-qPCR method

**DOI:** 10.1038/s41598-020-77119-6

**Published:** 2020-11-18

**Authors:** Zhiyuan Lin, Yongquan Chen, Yanling Lin, Huayue Lin, Hongwei Li, Xiaosong Su, Zanxi Fang, Jiajia Wang, Qingchun Wei, Jing Teng, Zhongying Zhang

**Affiliations:** 1 Center of Medical Laboratory, Xiamen Humanity Hospital Fujian Medical University, Xiamen, 361009 China; 2 Xiamen Key Laboratory of Biomarker Translational Medicine, Xiamen, 361009 China; 3 Clinical Laboratory Department, Xiamen Hospital of Traditional Chinese Medicine (Xiamen Hospital of T.C.M.), Xiamen, 361001 China; 4 Center of Clinical Laboratory of Zhongshan Hospital Affiliated to Xiamen University, Xiamen, 361004 China; 5 State Key Laboratory of Molecular Vaccinology and Molecular Diagnostics, School of Public Health, Xiamen University, Xiamen, 361102 China; 6 National Institute of Diagnostics and Vaccine Development in Infectious Disease, Xiamen University, Xiamen, 361102 China

**Keywords:** Biological techniques, Cancer, Molecular biology, Biomarkers, Molecular medicine, Oncology

## Abstract

miRNAs are expected to become potential biomarkers in the diagnosis and prognosis of Esophageal cancer (EC). Through a series of screening, miR-34a-5p, miR-148a-3p and miR-181a-5p were selected as EC-associated miRNAs. Based on AllGlo probe, a novel absolute quantitative RT-qPCR method with high sensitivity, specificity and accuracy was established for detecting miRNAs. Then the clinical significance of these 3 miRNAs was explored with 213 patients (166 cases with EC and 47 cases with benign diseases) and 170 normal controls. Compared with normal controls, the level of miR-34a-5p increased while miR-148a-3p and miR-181a-5p decreased in EC and benign patients (*P* < 0.001), and the level of miR-181a-5p in early EC patients was significantly lower (*P* < 0.001). According to logistic regression analysis, combined detection of miR-34a-5p, miR-148a-3p and Cyfra21-1 provided the highest diagnosis efficiency of 85.07% with sensitivity and specificity reaching 85.45% and 84.71%. Compared with preoperative samples, the level of miR-34a-5p decreased while miR-148a-3p and miR-181a-5p increased in postoperative samples (*P* < 0.001). Collectively, this first developed, novel absolute quantitative RT-qPCR method exhibits high application value in detecting miRNAs, miR-34a-5p, miR-148a-3p and miR-181a-5p may serve as potential biomarkers in the diagnosis and prognosis of EC, and miR-181a-5p probably could serve as a new biomarker for early EC.

## Introduction

Esophageal cancer (EC), one of the most common malignant tumors in the clinic, ranks the sixth leading cause of cancer mortality in the world with progressive dysphagia as the main clinical manifestation^1^. In China, EC is one of the highest morbidity and mortality cancers, about 258,000 new cases and 193,000 deaths of EC were reported in 2017^2^. There was evidence that patients with advanced EC or metastasis had a five-year survival rate of less than 20%, which was much lower than the concurrent survival rate of patients diagnosed with early cancer^3^. According to the National Health and Wellness Committee of China, currently, endoscopy combined with pathological examination is the golden standard of the diagnosis of EC^4^. However, the invasiveness, high cost and the missed diagnosis of early patients make it unable to be used as a common screening method for physical examination. Blood biomarkers, a kind of minimally invasive or non-invasive biomarker used in many kinds of cancers, can serve as the candidates for early diagnosis or the prognostic indicators of EC. At present, CEA, SCC and Cyfra21-1 are commonly used as esophageal tumor markers in clinical practice. However, due to the insufficient sensitivity and prognostic value, these tumor markers are difficult to replace other examinations to be the main auxiliary diagnostic indicators for EC^5^. Therefore, specific and sensitive blood markers are urgently needed for more effective diagnosis and treatment of EC.

MicroRNAs (miRNAs) , consisting of 21–25 nucleotides in length, are short single- stranded non-coding RNAs, of which the expression levels are related to tumor types and development stages^6–9^. Studies have shown that the expressions of miRNAs are involved in the developments, invasion and metastasis of several kinds of cancers, including colorectal cancer^10,11^, breast cancer^12,13^, lung cancer^14^, which are hopefully to become potential biomarkers in plasma in the diagnosis and therapy. However, researches on the expression of miRNAs in EC patients are few and the changes of the levels of miRNAs are still not very clear. Thus, considering the expression levels of miRNAs were different in the stages of development, invasion and metastasis of many other cancers, we decided to explore whether the abundance of miRNAs^15,16^ was changed in the plasma of EC patients and whether the miRNAs could serve as biomarkers for EC.

As is known, the common methods used to detect miRNAs include SYBR Green qPCR and TaqMan probe qPCR^17^. However, due to the restrictions in methodology or experimental cost, the specificity of SYBR Green qPCR is insufficient and the applicability in clinical practice of Taqman probe qPCR is limited. In order to solve these problems, a novel absolute quantitative RT-qPCR detection method by AllGlo probe was designed by us. The AllGlo probe is a new probe which was developed by ALLELOGIC BIOSCIENCES. It can overcome the shortcomings of traditional probes, greatly improving fluorescence gain and reducing Ct value. Thus, the AllGlo probe offers much higher sensitivity than those of traditional probes^18–20^. Compared with other probes, the TM value of the AllGlo probe will increase by 8–10 °C, leading to shorter sequence which is more suitable for the detection of small fragments of miRNAs. So we consider that the AllGlo probe probably owns the advantage of high sensitivity and specificity in detecting miRNAs.

In this study, firstly, we obtained three miRNAs (miR-34a-5p, miR-148a-3p, miR-181a-5p) from 13 candidate miRNAs^21–29^ in the plasma samples of discovery cohort. Then, we established a novel absolute quantitative RT-qPCR method after a series of performance verification experiments for detecting circulating miRNAs based on AllGlo probe. Finally, the clinical diagnosis significance of the screened miRNAs and their combined models in the diagnosis and prognosis of EC were explored.

## Results

### miR-34a-5p, miR-148a-3p and miR-181a-5p were selected as EC-associated candidate miRNAs

Through a series of screening procedures, three kinds of EC-associated candidate miRNAs (miR-34a-5p, miR-148a-3p, miR-181a-5p) were selected from 13 candidate miRNAs in plasma samples of discovery cohort (30 patients with EC and 30 normal controls) by SYBR GREEN (Table 1).

**Table 1:** Results of candidate miRNAs in discovery cohort.

NUM	Accession number	Micro RNA	P value
1	MIMAT0000416	hsa-miR-1-3p	0.154
2	MIMAT0000770	hsa-miR-133b	0.277
3	MIMAT0000435	hsa-miR-143-3p	0.454
4	MIMAT0000765	hsa-miR-335-5p	0.064
5	MIMAT0003294	hsa-miR-625-5p	0.066
6	MIMAT0000255	hsa-miR-34a-5p	0.033*
7	MIMAT0000243	hsa-miR-148a-3p	< 0.001***
8	MIMAT0000256	hsa-miR-181a-5p	< 0.001***
9	MIMAT0000419	hsa-miR-27b-3p	0.588
10	MIMAT0000244	hsa-miR-30c-5p	CT > 37
11	MIMAT0000510	hsa-miR-320a	0.485
12	MIMAT0000067	hsa-let-7f-5p	CT > 37
13	MIMAT0000414	hsa-let-7g-5p	CT > 37

*CT* cycle threshold.

*P < 0.05; ***P < 0.001.

### Establishment and evaluation of the novel absolute quantitative RT-qPCR method based on AllGlo probe for detecting miRNAs

**Figure 1:**
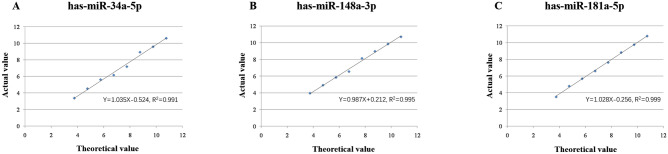
Linear range verification. (**A**) miR-34a-5p; (**B**) miR-148a-3p; C: miR-181a-5p. The linear equations of the three kinds of miRNAs were all R^2^ > 0.99, indicating that the detection method had a good linear relationship in the range of 5.6 × 10^3^–5.6 × 10^10^ copies/μL.

Using validated specific primers and designed probe sequence, the absolute miRNA quantitative RT-qPCR detection method based on AllGlo probe was constructed. AllGlo probe amplification products were sequenced, showing that all the miRNA PCR amplification products were specific. Compared with the SYBR Green method, the AllGlo probe detection method had a CT value of 1–2 cycles lower, which was of higher sensitivity. The stand curves (R^2^ > 0.99) were established for quantitative detection of the three kinds of miRNAs (miR-34a-5p, miR-148a-3p and miR-181a-5p). Moreover, both intra-assay and inter-assay variabilities were less than 5% (Table 2), indicating that the established detection method had good repeatability, stability and precision. Five different concentrations of standards were tested using a double-blind method to determine the correctness, the absolute deviations of the three kinds of miRNAs were all within acceptable ranges (≤ ± 0.4 log10) (Table 3). The correlation coefficients (R^2^) of the linear equations of the three miRNAs were all > 0.99, indicating that the detection method had a good linear relationship in the range of 5.6 × 10^3^ to 5.6 × 10^10^ copies/μL (Fig. 1). The LOD of miR-34a, miR-148a and miR-181a were 1906 copies/μL, 6202 copies/μL and 3332 copies/μL, respectively. Each of the eight high-concentration standards and the negative samples were cross-aligned and tested, the results showed that the negative specimens had no amplification curve, indicating that the detection was not affected by high-concentration samples. The methodological evaluation results proved that this absolute miRNA quantitative RT-qPCR detection method had high application value in scientific research and clinical promotion.

**Table 2:** Precision evaluation of the novel absolute quantitative RT-qPCR method.

miRNA	Concentration	Mean (log10)	SD			CV (%)		
			With-in run	With-in day	Total	With-in run	With-in day	Total
miR-34a-5p	Low concentration	3.25	0.07	0.11	0.13	2.06	3.41	3.90
	Middle concentration	5.49	0.06	0.07	0.09	1.15	1.22	1.66
	High concentration	8.95	0.03	0.08	0.08	0.34	0.86	0.85
miR-148a-3p	Low concentration	3.83	0.05	0.11	0.12	1.19	2.99	3.03
	Middle concentration	6.34	0.09	0.10	0.15	1.47	1.87	2.35
	High concentration	9.66	0.03	0.08	0.04	0.28	0.41	0.46
miR-181a-5p	Low concentration	3.49	0.06	0.10	0.11	1.69	2.88	3.23
	Middle concentration	5.92	0.06	0.12	0.11	0.97	1.70	1.82
	High concentration	9.70	0.03	0.04	0.08	0.31	0.86	0.84

**Table 3:** Accuracy evaluation of the novel absolute quantitative RT-qPCR method.

miRNA	Sample	Theoretical concentration (log 10)	Instrumental concentration (log 10)	Bias
miR-34a-5p	1	8.100421	8.209183	0.108762
	2	5.111464	4.756287	− 0.355177
	3	8.322270	8.513636	0.191366
	4	4.868426	4.808123	− 0.060303
	5	4.965336	5.157214	0.191878
miR-148a-3p	1	5.834048	5.955361	0.121313
	2	5.913229	5.952352	0.039123
	3	5.737138	5.852229	0.115091
	4	9.181236	9.446081	0.264845
	5	9.239227	9.555099	0.315872
miR-181a-5p	1	5.231054	5.222209	− 0.008845
	2	5.583237	5.237933	− 0.345304
	3	5.407146	5.009210	− 0.397936
	4	8.252751	8.455364	0.202613
	5	5.474092	5.428872	− 0.045220

### miR-34a-5p, miR-148a-3p and miR-181a-5p could be used to identify EC and benign esophageal diseases

**Figure 2:**
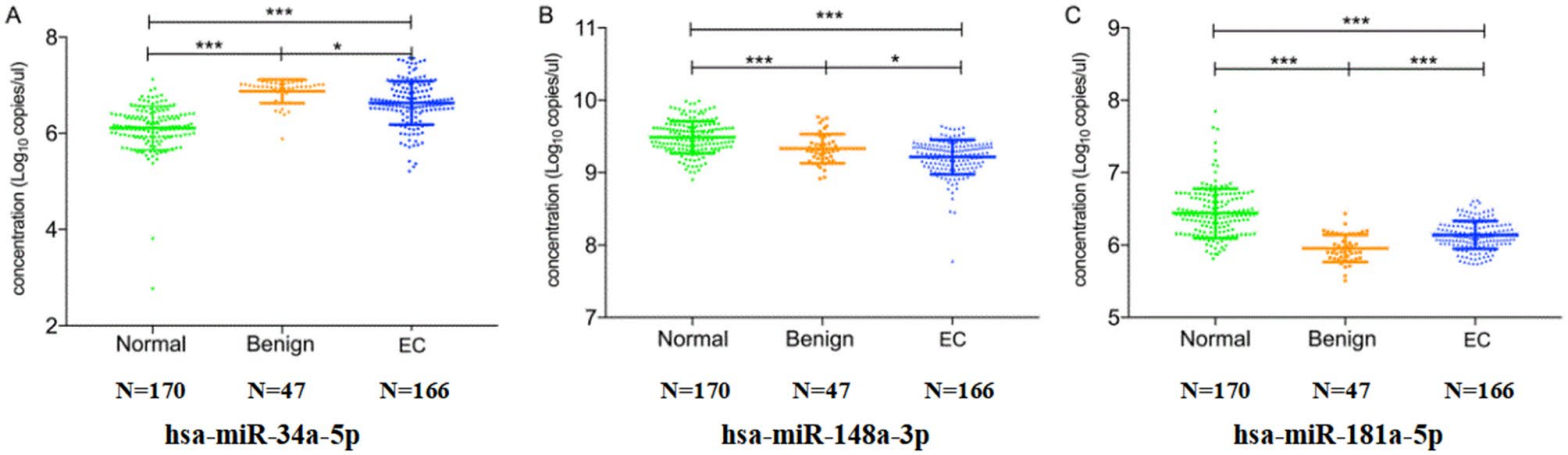
Expression levels of plasma miRNAs (miR-34a-5p, miR-148a-3p, miR-181a-5p) in EC patients, benign esophageal diseases patients and normal controls. (**A**) miR-34a-5p; (**B**) miR-148a-3p; (**C**) miR-181a-5p. The expression levels of miR-34a-5p, miR-148a-3p and miR-181a-5p in EC patients, benign esophageal diseases patients and normal controls were statistically significant (P < 0.001). The level of miR-34a-5p increased, while the levels of miR-148a-3p and miR-181a-5p decreased in EC patients. All data shown as log10 copies/μL. EC: esophageal cancer. *P < 0.05, **P < 0.01, ***P < 0.001.

The three kinds of differentially expressed plasma miRNAs were examined by AllGlo RT-qPCR in the validation tests with the plasma samples of 166 EC patients, 47 benign esophageal diseases patients and 170 normal controls. According to the examination results, The expression levels of miR-34a-5p, miR-148a-3p and miR-181a-5p in EC patients, benign esophageal diseases patients and normal controls were statistically significant (P < 0.001). Compared with normal controls, the level of miR-34a-5p increased, while the levels of miR-148a-3p and miR-181a-5p decreased in EC patients (Fig. 2).

### Evaluation of the clinical diagnostic value of the three miRNAs for EC

**Figure 3:**
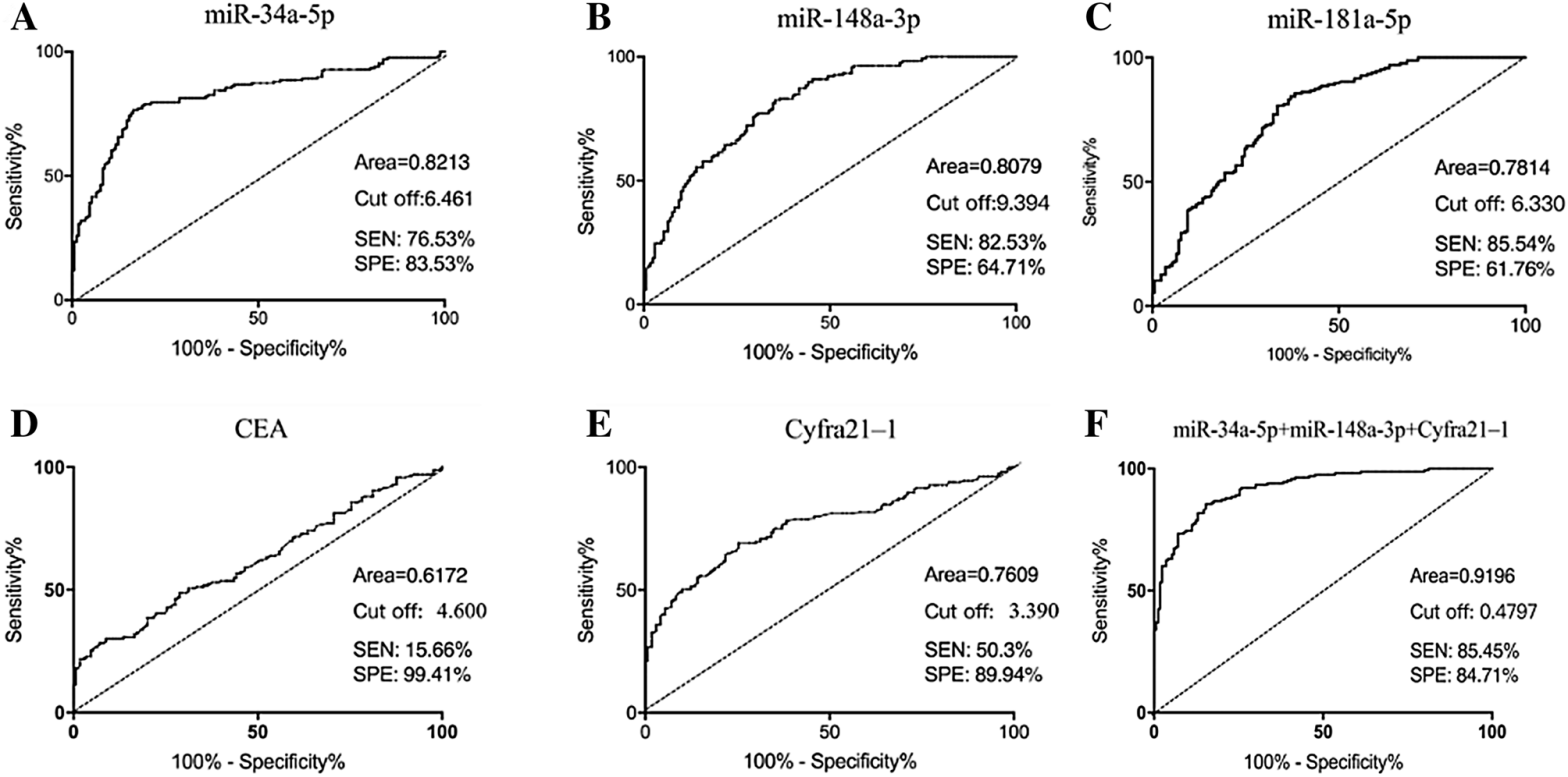
ROC curve analysis for distinguishing EC patients and normal controls. (**A**–**E**) The ROC curves of miR-34a-5p, miR-148a-3p, miR-181a-5p, CEA and Cyfra21-1 in distinguishing EC patients and normal controls. (**F**) The ROC curve of the panel of miR-34a-5p, miR-148a-3p and Cyfra21-1 in distinguishing EC patients and normal controls.

To evaluate the diagnostic efficiency of the three kinds of miRNAs, ROC curve was applied to analyze and find the appropriate cutoff value, respectively. As the levels of CEA and Cyfra21-1 in plasma are commonly used as auxiliary diagnostic markers for EC, the performance of the three kinds of miRNAs were compared with CEA and Cyfra21-1. ROC curve analysis showed that in distinguishing EC patients from normal controls, the areas under the curves(AUC) of miR-34a-5p, miR-148a-3p, miR-181a-5p were 0.8213, 0.8079, and 0.7814, respectively (Fig. 3A–C), while the areas of CEA and Cyfra21-1 were 0.6172 and 0.7609, respectively (Fig. 3D,E). The optimal cut-off values of miR-34a-5p, miR-148a-3p, miR-181a-5p, CEA and Cyfra21-1 were 6.461, 9.394, 6.330, 4.60 ng/mL and 3.39 ng/mL. At the optimal cut-off values, the sensitivity and specificity of miR-34a-5p were 76.53% and 83.53%, the sensitivity and specificity of miR-148a-3p were 82.53% and 64.71%, and the sensitivity and specificity of miR-181a-5p were 85.54% and 61.76%, while the sensitivity and specificity of CEA were 15.66% and 99.41%, and the sensitivity and specificity of Cyfra21-1 were 50.30% and 89.94%, indicating that the sensitivity of the three miRNAs were much higher than those of CEA and Cyfra21-1. Thus, miR-34a-5p, miR-148a-3p and miR-181a-5p in plasma could be complemented by the levels of CEA and Cyfra21-1 in plasma for the auxiliary diagnosis of EC. These findings validated the performance of miR-34a-5p, miR-148a-3p and miR-181a-5p as plasma markers for EC diagnosis.

In order to obtain higher diagnostic efficiency, the diagnostic efficiency of different combinations of the three kinds of miRNAs with CEA and Cyfra21-1 was examined by logistic regression analysis. As was shown in Table 4, the diagnosis efficiency of combinations were all higher than that of each marker used alone. In the 166 patients with EC, according to logistic regression analysis of the 3 kinds of miRNAs with CEA and Cyfra21-1, the regression coefficient of miR-181a-5p was − 0.306 (*P* = 0.627 > 0.05) in binary logistic regression (Forward) and the weight of CEA was so low that both of them were excluded. Based on economic benefit, the model of the panel of miR-34a-5p, miR-148a-3p and Cyfra21-1 which was of the highest diagnostic efficiency (85.07%) was chosen, and a mathematical diagnostic model through Logistics regression was obtained: Y = 2.774*miR-34a-5p − 5.536*miR-148a-3p + 0.881*Cyfra21-1. The ROC curve analysis showed that the AUC of the panel of miR-34a-5p, miR-148a-3p and Cyfra21-1 was 0.9196, with sensitivity and specificity reaching 85.45% and 84.71% (Fig. 3F). In the 67 patients with early EC, logistic regression analysis of the combination of miR-34a-5p, miR-148a-3p and Cyfra21-1 also showed that the combined model had a higher diagnostic efficiency (Table 4) than those of other panels. All of the above results indicated that the combined detection of the panel of miR-34a-5p, miR-148a-3p and Cyfra21-1 in plasma provided a higher diagnosis efficiency thereby further improving the accuracy of diagnosis.

**Table 4:** The combination diagnostic value between EC or early stages of EC and healthy control.

Group	Panel	Cutoff value	Sensitivity (%)	Specificity (%)	PPV (%)	NPV (%)	Diagnosis efficiency (%)
EC and control	miR-34a-5p, miR-148a-3p, miR-181a-5p	0.4545	87.95	78.82	80.22	87.01	83.33
	3 miRNAs, CEA, Cyfra21-1	0.5217	80.61	87.65	86.36	82.32	84.18
	miR-34a-5p, miR-148a-3p, CEA, Cyfra21-1	0.4793	83.03	85.29	84.57	83.82	84.18
	miR-34a-5p, miR-148a-3p, Cyfra21-1	0.4797	85.45	84.71	84.43	85.71	85.07
Early stages of EC and control	miR-34a-5p, miR-148a-3p, miR-181a-5p	0.2774	81.18	85.07	64.04	93.20	82.20
	3 miRNAs, CEA, Cyfra21-1	0.2544	86.47	86.36	71.25	94.23	86.44
	miR-34a-5p, miR-148a-3p, CEA, Cyfra21-1	0.3496	90.00	80.30	75.71	92.17	87.29
	miR-34a-5p, miR-148a-3p, Cyfra21-1	0.3504	89.41	81.82	75.00	92.67	87.29

### miR-181a-5p could serve for the diagnosis of early EC

**Figure 4:**
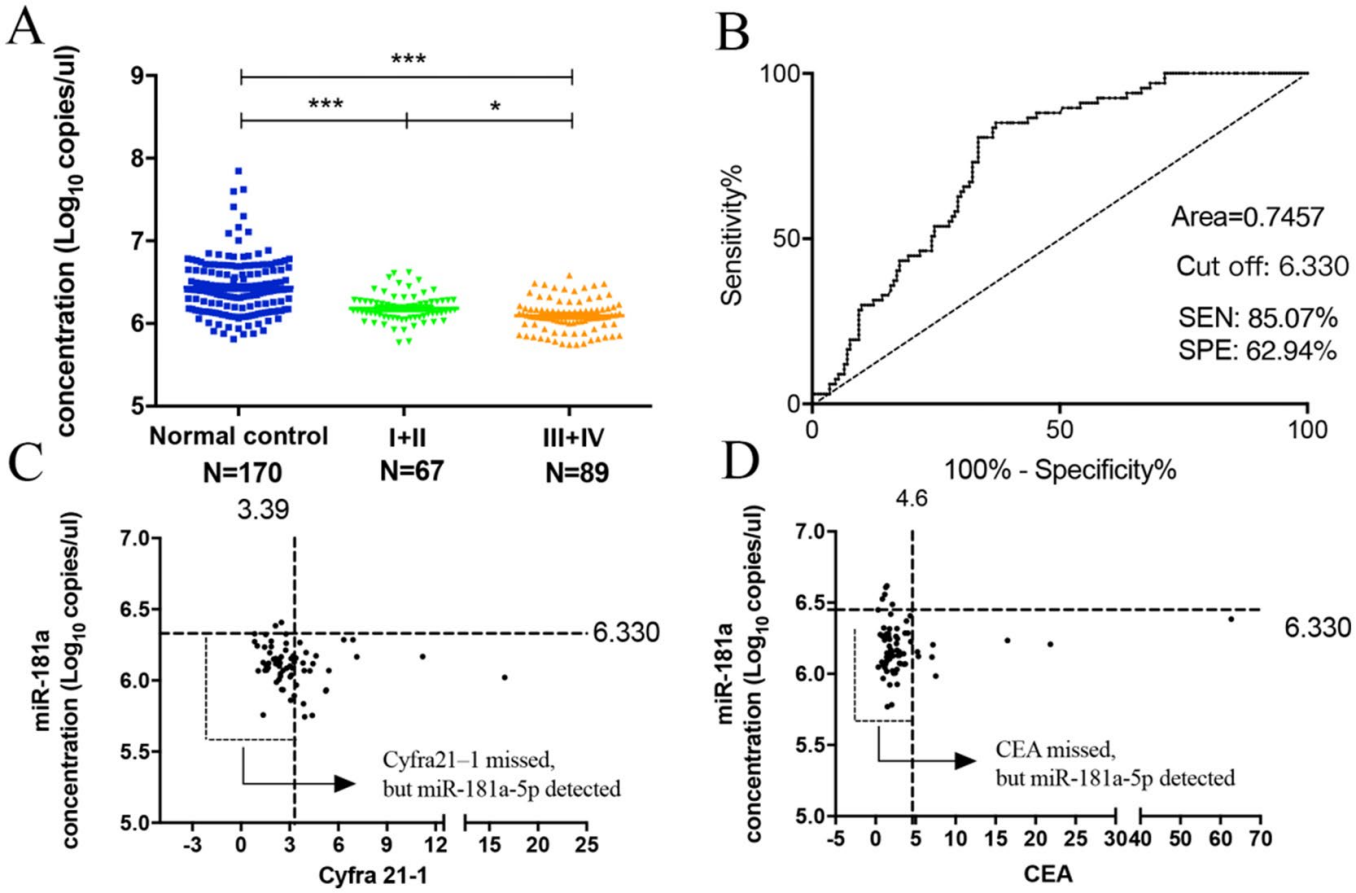
The diagnostic value of miR-181a-5p between early stage of EC patients and normal controls. (**A**) Expression of miR-181a-5p between normal controls and different stages (I + II, III + IV) of EC patients, showing that the level of miR-181-5p in early EC patients was significantly lower than that of normal volunteers. (**B**) ROC curve analysis of miR-181a-5p in distinguishing early EC patients and normal controls, demonstrating that miR-181a-5p possessed good diagnostic efficiency in distinguishing early EC patients and normal controls. (**C**,**D**) Two-parameter classification in detecting early stages of EC. In 67 patients with early EC, 36 patients that were missed by Cyfra21-1 alone (**C**) and 49 patients that were missed by CEA alone (**D**) were identified by miR-181a-5p. The cut-off values of miR-181a-5p, CEA and Cyfra21-1 were 6.330, 5.5 ng/mL and 3.39 ng/mL. *P < 0.05, **P < 0.01, ***P < 0.001.

To determine whether these three kinds of miRNAs could be used as tumor markers in the development and progression of EC, the correlations between the expression levels of miRNAs and clinical pathological features of patients were analyzed. No obvious differences were observed when EC patients were stratified by sex, age or other clinical features, whereas the expression levels of miR-181a-5p were different in patients with different TNM stages of EC (*P* = 0.0113, Table 5). In order to clarify whether the expression level of miR-181a-5p could be used for the diagnosis of early EC, we compared the expression levels of miR-181a-5p between early EC patients (156 cases, 10 EC patients with unclear TNM stage had been excluded from the total 166 ECpatients.) and normal controls. The expression level of miR-181a-5p in early EC patients was significantly lower than that of normal controls (P < 0.001, Fig. 4A). According to the ROC curve analysis of diagnostic efficiency of miR-181a-5p in early EC patients, the value of AUC was 0.7457, the diagnostic sensitivity and specificity were 85.07% and 62.94% with the optimal cutoff value of 6.330 (Fig. 4B). In 67 patients with early EC, 36 patients that were missed by Cyfra21-1 alone (Fig. 4C) and 49 patients that were missed by CEA alone (Fig. 4D) were identified by miR-181a-5p.

**Table 5:** The relationship between the expressions of miR-34a-5p/miR-148a-3p/miR-181a-5p and clinicopathological features of EC patients(166 cases).

Characteristics	Num	miR-34a-5p (log10 copies/μL)	P value	miR-148a-3p (log10 copies/μL)	P value	miR-181a-5p (log10copies/μL)	P value
**Age**							
≤ 60	68	6.544 ± 0.561	0.117	9.195 ± 0.261	0.521	6.145 ± 0.168	0.839
> 60	98	6.680 ± 0.351		9.227 ± 0.221		6.134 ± 0.205	
**Gender**							
Male	133	6.621 ± 0.456	0.444	9.219 ± 0.239	0.596	6.138 ± 0.188	0.915
Female	33	6.666 ± 0.446		9.193 ± 0.237		6.142 ± 0.202	
**Smoking**							
Positive	103	6.577 ± 0.486	0.040*	9.218 ± 0.252	0.456	6.138 ± 0.185	0.688
Negative	63	6.717 ± 0.381		9.206 ± 0.214		6.139 ± 0.199	
**Drinking**							
Positive	65	6.550 ± 0.546	0.057	9.225 ± 0.217	0.957	6.140 ± 0.181	0.887
Negative	101	6.682 ± 0.375		9.207 ± 0.251		6.137 ± 0.197	
**Type of CA**							
ESCC	141	6.648 ± 0.465	0.202	9.224 ± 0.244	0.086	6.073 ± 0.149	0.064
EAC	25	6.530 ± 0.368		9.156 ± 0.193		6.171 ± 0.085	
**Location**							
Upper	21	6.755 ± 0.406	0.366	9.231 ± 0.234	0.672	6.199 ± 0.229	0.569
Middle	78	6.643 ± 0.505		9.214 ± 0.215		6.130 ± 0.186	
Lower	67	6.576 ± 0.396		9.208 ± 0.226		6.130 ± 0.181	
Tumor size (cm)							
≥ 5	50	6.635 ± 0.400	0.827	9.196 ± 0.290	0.997	6.125 ± 0.185	0.564
< 5	116	6.628 ± 0.476		9.221 ± 0.212		6.144 ± 0.193	
**Differentiation**							
Poor	28	6.590 ± 0.482	0.915	9.216 ± 0.204	0.95	6.117 ± 0.157	0.056
Moderate	105	6.649 ± 0.457		9.212 ± 0.262		6.111 ± 0.182	
Well	26	6.565 ± 0.373		9.231 ± 0.188		6.212 ± 0.239	
Missing	7	6.748 ± 0.585		9.159 ± 0.163		6.131 ± 0.157	
**TNM stage**							
Tis, I	33	6.616 ± 0.462	0.176	9.256 ± 0.206	0.169	6.219 ± 0.176	0.011*
II	34	6.780 ± 0.413		9.236 ± 0.216		6.153 ± 0.166	
III	71	6.595 ± 0.476		9.172 ± 0.280		6.089 ± 0.199	
IV	18	6.604 ± 0.465		9.162 ± 0.128		6.117 ± 0.187	
Missing	10	6.462 ± 0.460		9.387 ± 0.121		6.209 ± 0.167	
**Pathology type**							
Fungating	43	6.704 ± 0.429	0.541	9.247 ± 0.229	0.532	6.170 ± 0.188	0.279
Erosion	23	6.631 ± 0.480		9.229 ± 0.184		6.172 ± 0.147	
Ulcer	51	6.629 ± 0.460		9.216 ± 0.173		6.109 ± 0.194	
Other	49	6.566 ± 0.457		9.175 ± 0.315		6.126 ± 0.204	

*P < 0.05.

In the process of exploring the ability of miR-181a-5p to distinguish early ECs from normal controls, we found that in the serial testing of miR-181a-5p and CEA, the specificity increased dramatically to 100%, while the sensitivity dropped to 11.1%. And in the parallel testing of miR-181a-5p and CEA, the sensitivity and specificity of the combination were 86.57% and 61.76%, which were similar to miR-181a-5p alone. Consistently, serial testing of miR-181a-5p and Cyfra21-1 increased specificity while reduced sensitivity, and parallel testing of miR-181a-5p and Cyfra21-1 failed to improve diagnostic efficiency. Indeed, the sensitivity of miR-181a-5p in the diagnosis of early EC was much higher than those of conventional tumor markers (CEA and Cyfra21-1). Our results provided evidence that the expression level of miR-181a-5p in plasma could be used to distinguish early EC patients from normal controls with clinically satisfactory sensitivity, which might be a new biomarker for early EC.

### The evaluation of miR-34a-5p, miR-148a-3p and miR-181a-5p serving as prognosis biomarkers for EC patients after surgery

**Figure 5:**
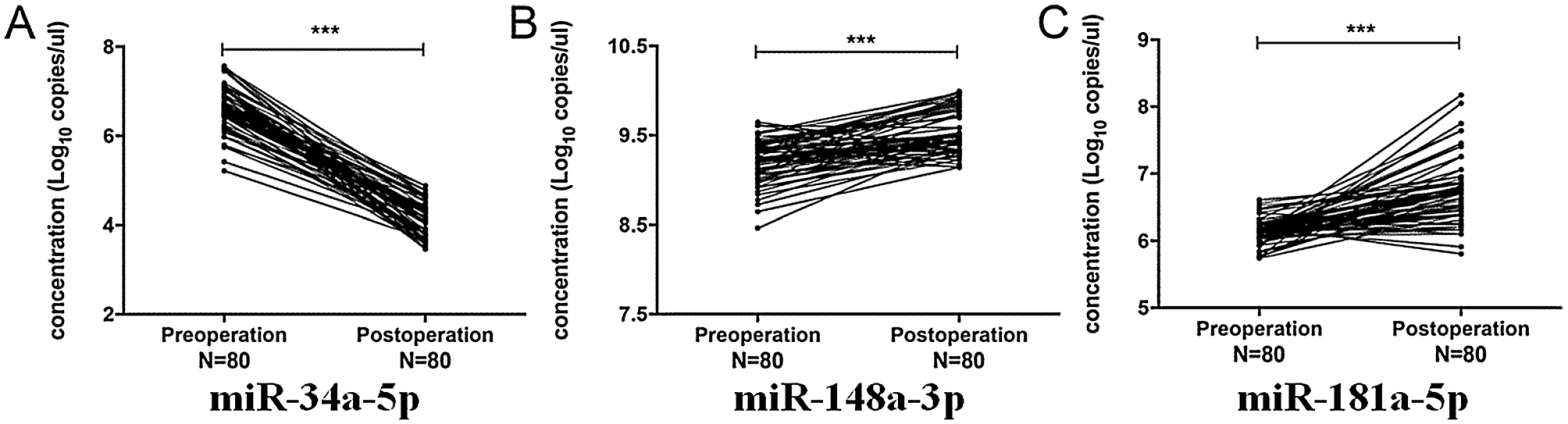
The changes of the levels of miR-34a-5p, miR-148a-3p, miR-181a-5p after the surgery (pre-operation vs. post-operation). All date shown as log10 copies/μL. *EC* esophageal cancer; ***P < 0.001.

In order to evaluate whether these three kinds of miRNAs could be used as prognosis biomarkers, the levels of the three kinds of miRNAs in preoperative and postoperative plasma samples of 80 EC patients who underwent esophagectomy were examined. The examination results showed that, compared with the preoperative samples, the level of miR-34a-5p significantly decreased, while the levels of miR-148a-3p and miR-181a-5p significantly increased in the postoperative samples (*P* < 0.001, Fig. 5). These results suggested that the levels of miR-34a-5p, miR-148a-3p and miR-181a-5p in plasma might be valuable predictors of postoperative prognosis for EC patients.

## Discussion

miRNAs have recently emerged as a novel class of gene expression regulators. Studies had shown that miRNAs were stabilized in the serum, of which the expression level was related to tumor types and development stages^18,19^. Thus, the circulating miRNAs may be a novel kind of potential biomarker for early diagnosis and clinical evaluation of EC patients. However, the mature miRNA is a kind of short RNA of 21-25nt, which easily interfered by homologous sequences and homologous miRNA sequences during the detection process. Also, many miRNAs are less abundant in circulation and difficult to be detected accurately^17^. At present, PCR detection methods for miRNA mainly include SYBR GREEN method and probe method. The SYBR GREEN method has a lower cost, but due to methodological restrictions, the specificity is poor. As for the most commonly used TaqMan probe, it has a long sequence and high experimental cost, which is difficult to design and accept^20^. These limitations make current PCR detections of miRNAs more difficult to promote clinically. The sensitivity and amplification efficiency of miRNA detection are very significant, and the stability and repeatability of the detection method are of great impact on the results, without which, even if the detection method is economically feasible, it is difficult to become a routine examination method in clinical.

Currently, AllGlo probe, the latest generation of quantitative fluorescent probe, has been applied in detecting H7N7, HPV and acute respiratory infection-associated virus. AllGlo probe has higher specificity and sensitivity than common methods as well as satisfactory cost effectiveness^30–32^. Since the AllGlo probe is shorter than other probes, the fluorophore will increase the TM value of the probe by 8–10 °C during the PCR reaction, making it more suitable for the detection of small fragment miRNAs. In the detection process by AllGlo probe, as long as there is a base mismatch in the amplification reaction, it will not produce a fluorescent signal, thereby greatly reducing the non-specific fluorescent signals and effectively resolving the interference of the miRNA precursors and homologous sequences. The advantages of this assay system overcome the problems of miRNAs detection and will promote miRNAs as novel tumor biomarkers in clinical diagnosis. In addition, the PCR method can also be applied to gene detection.

This study was the first to design an AllGlo-probe-based absolute quantitative RT-qPCR assay to identify and quantify miRNA, thus solving the above problems. Our method not only overcome the restrictions of miRNAs detection in plasma, but also was quantitative and convenient. The overall performance evaluation results of this method proved that the detection method was stable, accurate and sensitive with no contamination or cross-influence, which had significant application value in scientific research and clinical diagnosis. Compared with the SYBR Green qPCR, the AllGlo qPCR method had a higher sensitivity and a wider linear range (10^3^–10^10^ copies/μL), meaning that we could easily detect the miRNAs which were expressed in low abundance in the circulation. The levels of miRNAs in some body fluids such as urine, cerebrospinal fluid and exosome are much lower than those in serum or plasma, however studies had shown that they have great significance in the process of cancer development or some other diseases^33–35^.

The established system was initially used to explore the diagnostic efficiency of plasma miRNAs through expanded sample size, which also provided evidence of the reliability of the method. We found that the expression level of miR-34a-5p increased, whereas the expression levels of miR-148a-3p and miR-181a-5p decreased in the plasma of EC. The results indicated that the plasma levels of miR-34a-5p, miR-148a-3p and miR-181a-5p could serve as biomarkers for EC diagnosis. The difference in the expression level of miRNA in benign diseases and EC may be related to the regulation of the development of EC by miRNA. According to the studies of Wang et al. and Han et al^25,36^, miR-34a-5p could inhibit proliferation, migration, invasion and epithelial-mesenchymal transition in Esophageal Squamous Cell Carcinoma by targeting lymphoid enhancer-binding factor 1 and suppressing the Hippo-YAP1/TAZ signaling pathway, and The lncRNA CRNDE could promote colorectal cancer cell proliferation and chemoresistance via miR-181a-5p-mediated regulation of Wnt/β-catenin signaling. Perhaps this is why the expression of miR-34a-5p was upregulated and the expression of miR-181a-5p was deregulated in benign disease compared to EC patients, and there may also be some other regulatory mechanisms.

Our results also showed that combination of the three kinds of miRNAs could be used as a more comprehensive indicator of tumor detection compared to Cyfra211 and CEA. In particular, we observed significant differences in the expression of miR-181a-5p in EC patients at different stages of development and normal controls. The expression level of miR-181a-5p was lower in early EC patients than that in normal controls with the sensitivity 85.07%, indicating that miR-181a-5p could be used as a biomarker for early diagnosis of EC. In order to obtain the optimal diagnostic performance, we combined clinical indicators (CEA, Cyfra21-1) with these three kinds of miRNAs to construct a diagnostic mathematical model. After the indicators were combined and analyzed by logistic regression, the mathematical formula was constructed according to the different weights in the diagnosis process, of which the diagnostic AUC was up to 0.9196, and the sensitivity and specificity were up to 85.45% and 84.71%, respectively. Similar to the Roman index, this mathematical formula has a strong practicality, with which we can evaluate the risk of EC based on the examination results of miR-34a-5p, miR-148a-3p and Cyfra21-1, reducing the false negative rate, thus improving the diagnosis efficiency of EC.

In this study, when compared with the preoperative samples, the level of miR-34a-5p significantly decreased, while the levels of miR-148a-3p and miR-181a-5p significantly increased in the postoperative samples (*P* < 0.001, Fig. 5). These results suggested that the levels of miR-34a-5p, miR-148a-3p and miR-181a-5p in plasma might be valuable predictors of postoperative prognosis for EC patients. As for the mechanism of the change of miRNA expression before and after surgery, it was a relatively complicated process. Since the expression level of miRNA is closely related to tumor proliferation, migration, invasion and epithelial-mesenchymal transition processes^36^, when the tumor tissue of EC patients was removed by surgery, the growth of the tumor was basically stagnant, and the inhibitory effect of miR-34a-5p was also reduced. Through this study, we found that the expression level of miR-34a-5p was positively correlated with the development of tumor tissue to a certain extent. In addition, recent studies also showed that miR-34a-5p played an important role in the immune system, especially in the chemotherapy process of patients with malignant tumors. Ebrahimiyan^37^, et al.’s study showed that altered expression of survivin, regulated by miRNAs, such as miR-34a-5p, may result in apoptosis resistance and auto-reactivity in lymphocytes from patients and have important roles in systemic sclerosis pathogenicity. Zuo^38^ et al.’s study demostrated that miR-34a-5p negatively regulated the expression of PD-L1 by targeting its 3′-untranslated region and miR-34a-5p/PD-L1 axis regulated cis-diamminedichloroplatinum (DDP) chemoresistance of ovarian cancer cells. Also, Luo^39^ et al.’s study discovered that TP73‑AS1 contributed to proliferation, migration and DDP resistance but inhibited apoptosis of non-small cell lung cancer cells by upregulating TRIM29 and sponging miR‑34a‑5p. These studies have directly or indirectly shown that miR-34a-5p has an important regulatory role in the function of the body’s immune system. As for the association between miR-34a-5p and smoking, it is not very clear at present. Sui^40^ et al.’s study showed that non-smoking lung adenocarcinoma patients, compared to smokers, had different characteristics in terms of somatic mutation, gene, and miRNA expression and the microenvironment, indicating a diverse mechanism of oncogenesis. In our study, probably because the sample size was relatively small, the difference between miR-34a-5p and smoking showed a statistical difference, but not very obvious. Regarding the relationship between miR-34a-5p and smoking, more research may be needed to prove.

In summary, the novel absolute quantitative RT-qPCR method based on AllGlo probes designed to detect miRNAs possesses the advantage of high stability, accuracy and sensitivity. It has great application value in scientific research and clinical diagnosis. Meanwhile, by using this developed method, we identified that miR-34a-5p, miR-148a-3p and miR-181a-5p may serve as novel noninvasive biomarkers for EC diagnosis and prognosis, especially, miR-181a-5p probably could be used as a new biomarker for early EC. However, this study is still in its infancy, requiring more different types of samples and different kinds of miRNAs to ultimately optimize the detection system and method. We will continue to expand the sample size of EC, especially the patient's postoperative samples and samples during postoperative treatment. Simultaneously, information on the treatment, prognosis, and survival of EC patients will be collected, in order to further study, the specific role of these miRNAs in the prognosis of EC. Additionally, we will take follow-up studies to determine whether the plasma levels of these three kinds of miRNAs can predict recurrence/metastasis of EC.

## Materials and methods

This study had been approved by the Ethics Review Board for human studies of Zhongshan Hospital Affiliated to Xiamen University, and all the participants had signed informed consents before the study began. All the methods in this study were performed in accordance with the relevant guidelines and regulations of the Fourth Edition of Clinical Laboratory Operation Procedures in China.

### miRNAs screening

Candidate miRNAs were selected based on the following rules: (1) the up-regulated or down-regulated miRNAs in cancers compared with control groups from the miRCancer database (https://mircancer.ecu.edu/search.jsp), (2) the miRNAs with potential clinical value which hadn’t been detected or analyzed in EC patients; and (3) the Ct value in plasma which was less than 30.

### Primers design

The sequences of candidate miRNAs were obtained from miRBase (https://www.mirbase.org/). Through Primer Premiers 5.0 and BLAST sequence comparison, the best specific primers and probes were selected and then synthesized by GENEJUE (Xiamen, China) and YiYue (Shanghai, China), respectively.

### miRNAs extraction

According to the manufacturer’s protocol of miRcute miRNA isolation kit (TIANGEN, Beijing, China), firstly, the miRNAs in plasma were extracted. Secondly, the purified miRNAs were eluted in 30 μL RNase-free water. Thirdly, the purity and concentration of the purified miRNAs were evaluated by NanoDrop 2000 Spectrophotometer (Thermo Fisher Scientific). Finally, the isolated miRNA samples were quickly aliquot and immediately stored at − 80 °C until use.

### Reverse transcription

The reverse transcription (RT) was carried out in a 20 μL reaction mixture containing 40 U/μL Moloney murine leukemia virus (MMLV) reverse transcriptase (Promega, WI, USA), 20 U/μL ribonuclease inhibitor (TAKARA, Dalian, China), 40 nmol dNTP mix (TAKARA, Dalian, China), 10 μmol stem loop RT primer (SANGON, Shanghai, China), 2 μL template miRNA, 4 μL MMLV RT buffer (Promega, WI, USA) and 11.6 μL RNAase-free water (Promega, WI, USA). After being mixed gently, the reaction mixtures were incubated at 25 °C for 5 min, 42 °C for 60 min and then 70 °C for 15 min. The final cDNA products were stored at − 30 °C until use. Each sample was repeated twice and RNase-Free water, instead of template miRNA, was used as negative control in RT.

### Verification of the sensitivity and specificity of the primer and probe by SYBR Green PCR

To determine the sensitivity and specificity of the primer, the SYBR Premix Ex Taq II (Tli RNaseH Plus) (TAKARA, Dalian, China) was used for real time PCR. The 20 μL reaction mixtures contained 10 μL SYBR Premix Ex Taq II (Tli RNaseH Plus) (2×) mixed with 0.4 μL Rox [ROX Reference Dye II (50×)], 0.2 μM forward and reverse primers respectively, 2 μL cDNA and 6.8 μL DEPC-water. The SYBR Green PCR was performed on an Applied Biosystems 7500 Sequence Detection System (ABI, USA) under the following PCR conditions: 95 °C for 30 s, followed by 50 cycles of 95 °C for 5 s, 60 °C for 30 s. To determine the sensitivity and specificity of the probes, the 20 μL reaction mixtures contained 10 μL qPCR Mast Mix (2×) mixed with 1 μM probe, 0.2 μM forward and reverse primers respectively, 2 μL cDNA.

### Verification of the sensitivity and specificity of the absolute quantitative RT-qPCR

The target miRNAs, used as templates which were synthesized by GENEJUE (Xiamen, China), were diluted by RNase-free water. The PCR reaction was performed on the ABI7500 to calculate the expansion efficiency and standard curve equation. In order to evaluate the precision of ALLGLO RT-PCR established in this study, the positive controls with three concentrations used as the templates were tested for consecutive 5 days, four times a day. According to the file EP9-A2, intra-CV value, daytime CV value and total CV value were calculated with the equation of CV = standard deviation/mean × 100%. A CV value < 5% is required. In order to check the correctness of the system, five different concentrations of standards were tested by a double-blind method and the mean, standard deviation and bias were calculated (EP9-A2). The high-concentration standard was serially diluted to eight different concentrations of standards and each sample was repeatedly measured twice, then the linear range and linear correlation coefficient were calculated with R^2^ > 0.95 required (EP6-A). In order to determine the limit of detection (LOD), the middle-concentration standard was diluted to the limit of the range used as template in PCR and the diluted standards were repeatedly measured 20 times to determine the LOD of this method. In order to determine the contamination carrying rate of the detection method, a total of eight high-value standards and negative specimens were interspersed.

### Samples and clinical pathological data collection

The plasma samples were collected in the Center of Clinical Laboratory of Zhongshan Hospital Affiliated to Xiamen University from April 2016 to February 2018. A total of 213 patients who were diagnosed with esophageal related diseases (166 cases with EC and 47 cases with benign diseases) and 170 normal controls who were matched with age and gender were recruited in this study. All the patients were pathologically diagnosed with surgical specimens or biopsies. Preoperative plasma samples were collected before esophagectomy and postoperative plasma samples were collected in the second week after esophagectomy from 80 of the 166 EC patients. None of the normal controls had prior history of any major illness. The clinic pathological characteristics of all the patients and controls were presented in Table 4. Tumor stages and differentiation levels were determined using the TNM staging classification system published by American Joint Committee on Cancer (AJCC) in 2009. Before any treatment, 2 mL venous blood sample anticoagulated with sodium citrate from each participate was collected and immediately centrifuged to get the plasma which were then stored at − 80 °C. This study was approved by the Ethics Committee of Zhongshan Hospital Affiliated to Xiamen University, and written informed consents were provided by all the participants.

### Verification and validation of the selected miRNAs as biomarkers

The abundance of miRNAs was detected by the absolute quantification RT-qPCR method based on AllGlo probe which was designed above. The concentrations of CEA and Cyfra21-1 in plasma were detected by Roche Cobas e601 system based on the principle of electrochemical luminescence. The cut-off point of CEA is 4.6 ng/mL and the detection limit is 0.20 ng/mL with a CV < 5%. The cut-off point of Cyfra21-1 is 3.39 ng/mL and the detection limit is 0.10 ng/mL with a CV < 5%. Samples were randomly detected blindly by trained clinical laboratory technicians before interpretation.

### Statistical analysis

The nonparametric Mann–Whitney *U* test was performed to compare the miRNA expression between the cancer patients and the normal controls, and Kruskal–Wallis test was used in more than two groups. Wilcoxon signed-rank test was used to determine the relative expression between pre and postoperation. The Mann–Whitney *U* test and the Kruskal–Wallis test were used to evaluate the correlations between the results of the miRNA expression and the clinicopathological parameters. Two-tailed P-value of < 0.05 was considered statistically significant. ROC curve was used to analyze the diagnostic sensitivity, specificity and diagnostic efficiency. The best diagnostic efficiency is judged by Youden index (sensitivity + specificity − 1).
